# Lysine supplementation is not effective for the prevention or treatment of feline herpesvirus 1 infection in cats: a systematic review

**DOI:** 10.1186/s12917-015-0594-3

**Published:** 2015-11-16

**Authors:** Sebastiaan Bol, Evelien M. Bunnik

**Affiliations:** Department of Botany and Plant Sciences, University of California, Riverside, 900 University Avenue, Riverside, CA 92521 USA; Department of Cell Biology and Neuroscience, University of California, Riverside, 900 University Avenue, Riverside, CA 92521 USA

**Keywords:** Lysine, FHV-1, Feline herpesvirus 1, Systematic review, Evidence-based medicine, Arginine, Antagonism, Upper respiratory disease, Conjunctivitis

## Abstract

**Background:**

Feline herpesvirus 1 is a highly contagious virus that affects many cats. Virus infection presents with flu-like signs and irritation of ocular and nasal regions. While cats can recover from active infections without medical treatment, examination by a veterinarian is recommended. Lysine supplementation appears to be a popular intervention (recommended by > 90 % of veterinarians in cat hospitals). We investigated the scientific merit of lysine supplementation by systematically reviewing all relevant literature.

**Methods:**

NCBI’s PubMed database was used to search for published work on lysine and feline herpesvirus 1, as well as lysine and human herpesvirus 1. Seven studies on lysine and feline herpesvirus 1 (two in vitro studies and 5 studies with cats), and 10 publications on lysine and human herpesvirus 1 (three in vitro studies and 7 clinical trials) were included for qualitative analysis.

**Results:**

There is evidence at multiple levels that lysine supplementation is not effective for the prevention or treatment of feline herpesvirus 1 infection in cats. Lysine does not have any antiviral properties, but is believed to act by lowering arginine levels. However, lysine does not antagonize arginine in cats, and evidence that low intracellular arginine concentrations would inhibit viral replication is lacking. Furthermore, lowering arginine levels is highly undesirable since cats cannot synthesize this amino acid themselves. Arginine deficiency will result in hyperammonemia, which may be fatal. In vitro studies with feline herpesvirus 1 showed that lysine has no effect on the replication kinetics of the virus. Finally, and most importantly, several clinical studies with cats have shown that lysine is not effective for the prevention or the treatment of feline herpesvirus 1 infection, and some even reported increased infection frequency and disease severity in cats receiving lysine supplementation.

**Conclusion:**

We recommend an immediate stop of lysine supplementation because of the complete lack of any scientific evidence for its efficacy.

**Electronic supplementary material:**

The online version of this article (doi:10.1186/s12917-015-0594-3) contains supplementary material, which is available to authorized users.

## Background

Feline herpesvirus 1 (FHV-1) infection is a common problem in cats, with reports of active infection rates ranging from 5 to 20 % [1–3]. Discrepancies in reported prevalences may be caused by, among others, differences in diagnostic methodologies, study populations and housing situations.

During active infection, cats may present with frequent sneezing, inflammation of the eyes and nasal mucous membranes, congestion, ocular and nasal discharge, facial or nasal dermatitis and may suffer from depression, lethargy and loss of appetite. The virus can be transmitted to other cats via ocular, oral and nasal secretions. After recovery, the virus will reside in a cranial nerve and the cat will be a lifelong carrier. Viral reactivation can be induced by a compromised immune system as the result of comorbidity, or by mental stress caused by for example moving the cat.

Although most cats will fully recover from the disease manifestations caused by active FHV-1 infection with tender loving care and may not require any medical treatment, it is recommended to have cats seen by a veterinarian when signs of active infection present. Supplementing cat food with the amino acid lysine has been advocated for the prevention and treatment of FHV-1 infection [4]. It is believed that excess dietary lysine will reduce viral shedding, thereby reducing the risk of infecting other cats in multi-cat households, shelters and catteries, and that excess lysine has a beneficial effect on recurrent outbreak frequency and the progression of disease manifestations. To determine how widespread this recommendation for the use of lysine is, we performed a small survey among 68 cat hospitals in the United States (57), Australia (7) and the United Kingdom (4). After having confirmed that the veterinarians were seeing and treating cats on a regular basis, they were asked if they recommend lysine supplementation for cats infected with feline herpesvirus 1 and if cat guardians can buy lysine in their clinic. We received 23 replies, 18 from cat hospitals in 12 different states of the United States, 3 in Australia and 2 in the United Kingdom. In 91 % (21 out of 23) of the cat hospitals, the veterinarian(s) recommend(s) lysine supplementation for cats infected with FHV-1, and in 87 % of the cat hospitals, lysine supplements are sold.

Given the extensive use of lysine supplementation for the treatment or prevention of FHV-1 infection in cats, we sought to evaluate the scientific basis for this recommendation. In this systematic review, we discuss details of the proposed mechanism of action, as well as the results from in vitro studies and clinical trials. Research on human herpesvirus 1 (HHV-1) is also reviewed in detail, since results from these experiments underlay all the work done on FHV-1.

## Methods

**Fig. 1:**
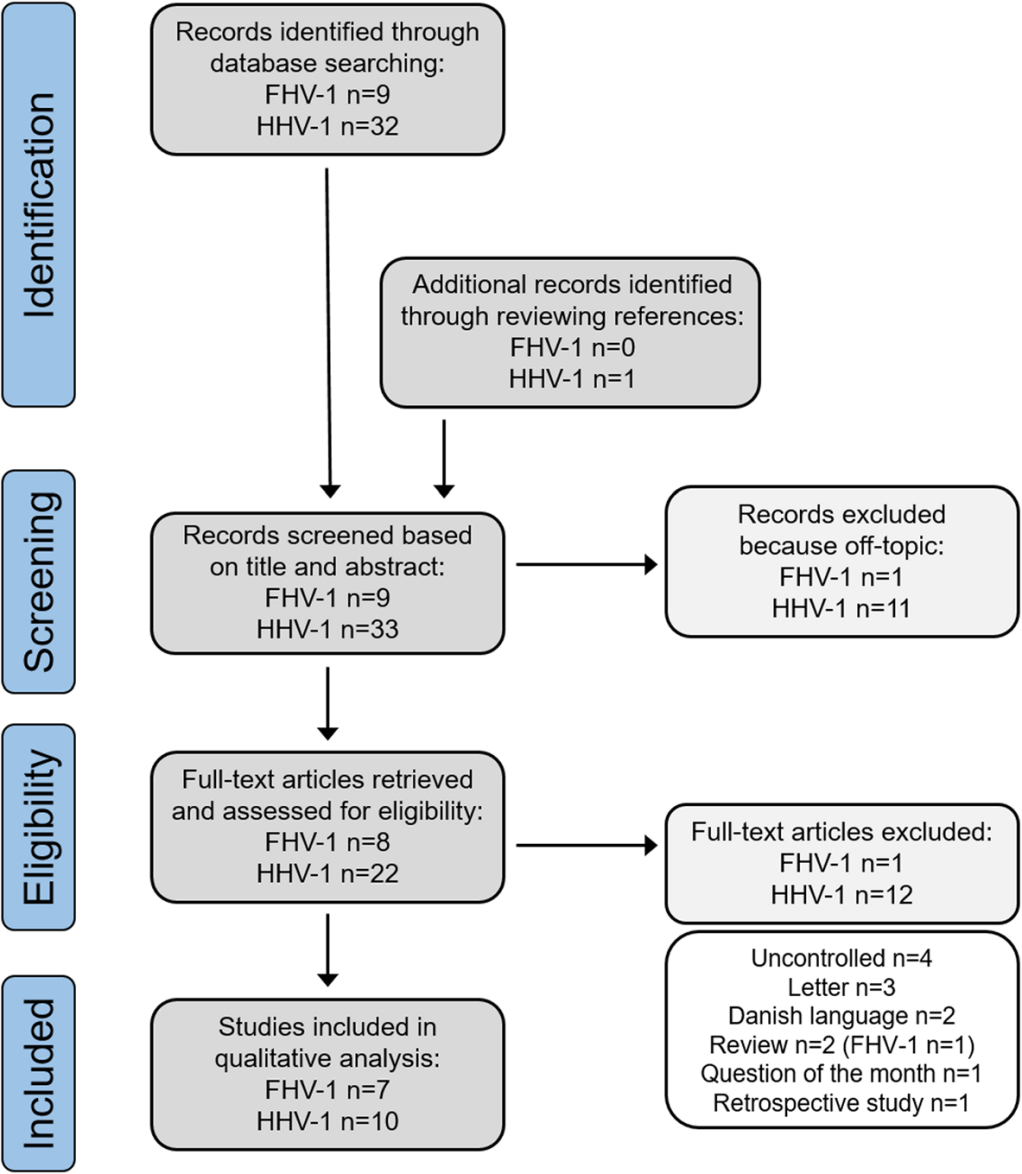
PRISMA flowchart for the selection of reviewed publications

This systematic review was performed in accordance with the PRISMA (Preferred Reporting Items for Systematic Reviews and Meta-Analyses) guidelines [5, 6] (see Additional file 1). NCBI’s PubMed database was searched for publications using the search ((lysine) AND (feline herpesvirus OR FHV-1)) in April 2015, without any restrictions. For work on lysine and HHV-1 the PubMed database was searched for publications using the search ((lysine [ti]) AND ((herpes simplex [ti]) OR (HSV [ti]) OR (human herpesvirus [ti]) OR (HHV [ti]) OR (herpes labialis [ti]))) in April 2015, without any restrictions. A total of 42 publications were screened on title and abstract, 9 for FHV-1 and 33 for HHV-1 (see Fig. 1). Full-text articles were retrieved for in vitro studies investigating the effect of lysine on the replication of FHV-1 or HHV-1, and for clinical trials evaluating the effect of oral lysine supplementation on the prevention or treatment of FHV-1 or HHV-1 infection. Twelve publications did not meet this criterion and were considered off-topic. Of the remaining 30 publications, 17 were included in the qualitative analysis: 2 in vitro studies with FHV-1 [7, 8], five clinical trials with cats [9–13], three in vitro studies with HHV-1 [14–16] and 7 clinical trials with humans [17–23]. Thirteen of the 30 publications were considered ineligible. Four because the studies were uncontrolled (all HHV-1), six because they were a letter, review or similar publication type (1 review for FHV-1), one because it was a retrospective study (HHV-1), and 2 because the articles were written in the Danish language (HHV-1). Both these publications, however, do not contain data that was not published in other manuscripts by the same authors, which are included in this systematic review (Dr. Milman, personal communication).

For the in vitro studies, data was extracted on cell type, multiplicity of infection (number of viral particles per cell), cell viability, culture time, arginine concentration in the medium and lysine concentration in the medium, when available. The outcome measure was the difference in replication kinetics of FHV-1 or HHV-1 comparing various lysine concentrations. For the clinical trials, data was extracted on study design, population size, study length, dose and frequency of lysine supplementation as well as plasma arginine and plasma lysine concentrations, when available. The measures of treatment effect were differences in frequency of initial infection, viral shedding, frequency and severity of clinical signs, and healing time in the studies with cats, and herpetic outbreak recurrence frequency, severity and healing time in the human clinical trials, comparing lysine supplementation to placebo.

For the amino acid composition analysis, amino acid sequences of coding regions of Felid herpes virus 1 (NCBI reference sequence NC_013590.2) and Human herpes virus 1 (NCBI reference sequence NC_001806.1) were downloaded from Genbank. Amino acid sequences of all known genes or novel gene predictions of the human genome (version GRCh38.p2) and the cat genome (version 6.2) were obtained from Ensembl (release 80) [24]. Amino acid compositions were calculated for individual protein sequences, as well as for all combined protein sequences of each organism.

## Results and discussion

### Mechanism

It is believed that lysine itself does not have antiviral properties, but that it affects replication of HHV-1 and FHV-1 by lowering arginine concentrations. Here, we discuss (1) the proposed antagonistic effects of lysine on arginine, and (2) how lowering of arginine concentrations is suggested to affect herpesvirus replication. We conclude with (3) a section discussing the importance of arginine for the cat.

#### Lysine-arginine antagonism

The effect of excess lysine on arginine levels has been addressed in several organisms. Chicks fed a diet high in lysine, but containing normal arginine levels, showed reduced growth, increased plasma lysine levels and decreased arginine in plasma and tissue [25–27]. Supplementary arginine in the diet reversed the observed signs of excess lysine, supporting the existence of lysine-arginine antagonism. A similar negative effect of excess dietary lysine on growth was obtained in a study with growing dogs [28] and weanling rats [29], although plasma arginine levels remained unaffected. Again, growth problems in these animals were neutralized by feeding extra arginine. Experiments with weaned pigs showed no growth problems and no changes in arginine levels when fed lysine-rich food [30], suggesting that the antagonism is species-specific. Lysine-arginine antagonism has not been well-studied in humans, but patients with hyperlysinemia, due to a mutation in a gene coding for a protein involved in the breakdown of lysine, do not show lowered plasma arginine levels [31], suggesting that humans may not be affected by an antagonism between these amino acids.

Fascetti et al. studied the effect of high levels of lysine in cat food on plasma arginine levels in cats [32]. The basal diet for the control group contained 11 g lysine and 13 g arginine per kg food, meeting the minimal arginine requirement of 10 g per kg food for an adult cat. Different groups of cats were fed experimental diets containing 36, 61, 86, 111 or 131 g lysine per kg food. Higher lysine concentrations in the diet were associated with reduced food intake. Plasma arginine levels remained unchanged at any of the lysine concentrations tested. Based on these results we conclude that, in the cat, excess lysine in the diet is unable to lower arginine levels in plasma. Similarly, Ball et al. concluded that there is no lysine-arginine antagonism in cats [33]. However, only adult cats were studied, and arginine levels in plasma, not in tissue, were measured. Growing animals appear to be more susceptible to the effects of excess lysine than fully grown animals [34]. It may be possible that, like in puppies [28], excess dietary lysine causes growth depression and other clinical signs of arginine deficiency in kittens, without affecting plasma arginine levels. Although research has been done on the requirements of lysine and arginine in young cats, the arginine requirement was established after the requirement for lysine was determined, and the effects of excess lysine on arginine requirements were therefore not investigated [35].

**Fig. 2:**
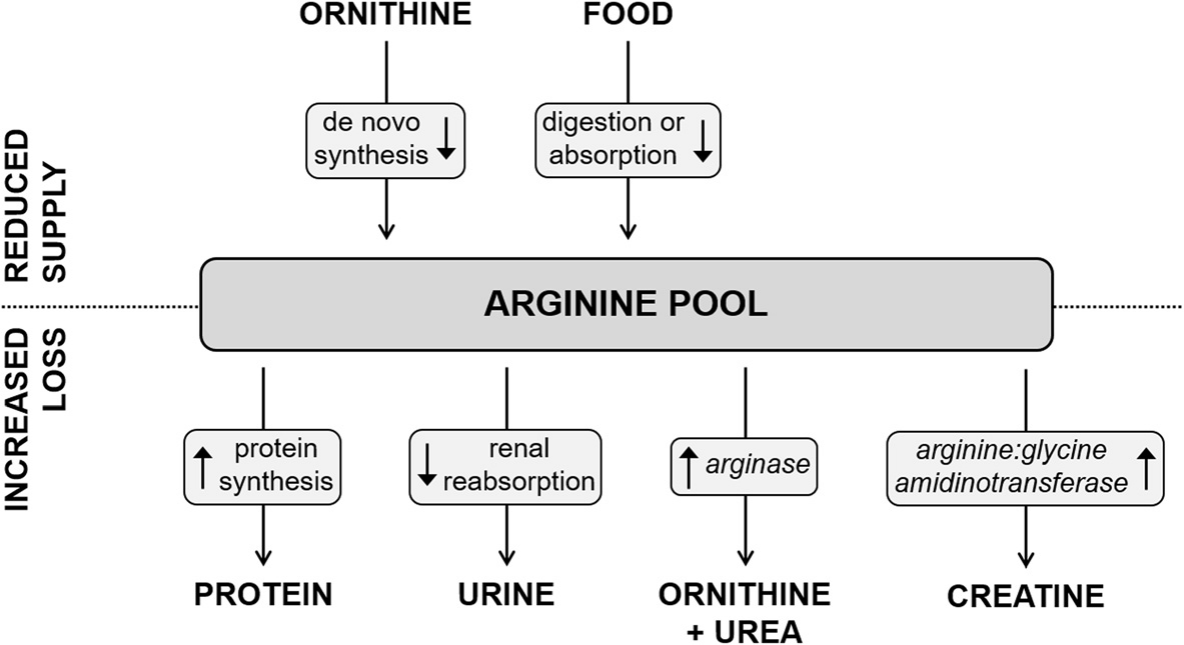
Mechanisms by which lysine may lower plasma and tissue arginine levels. The top part of the figure shows the mechanisms that may decrease arginine levels due to a reduction in the supply to the arginine pool, while the bottom part depicts the mechanisms that may lower arginine levels due to increased loss of the amino acid from the pool

Several studies have tried to elucidate the mechanism by which lysine affects arginine levels. In theory, the decrease in plasma arginine can be caused by (1) inhibition of the absorption of arginine in the intestine, (2) inhibition of de novo synthesis of arginine, (3) increased protein synthesis, (4) increased metabolism of arginine, or (5) increased excretion of arginine (see Fig. 2). Each of these mechanisms will be addressed in more detail below.

Lysine and arginine use the same systems for transportation across the cell membrane of intestinal and other cells (reviewed in [36]). However, it is unlikely that excess lysine prevents the absorption of arginine, since both amino acids can be absorbed throughout the entire length of the small intestine [37]. Furthermore, while another amino acid, leucine, inhibits the absorption of arginine in the intestine, excess dietary leucine in chicks was unable to induce signs of arginine deficiency [37]. In addition, supplementing small amounts of arginine to a diet high in lysine reversed the adverse effects of lysine [25]. Finally, results from some experiments suggested that lysine may reduce the availability of arginine in the intestine by inhibiting the activity of carboxypeptidase B or trypsin, digestive enzymes that cleave proteins and peptides at arginine and lysine residues [27, 29]. However, the authors concluded it is unlikely that this significantly reduces arginine utilization. This conclusion is supported by the observation in chicks that excess lysine also resulted in growth depression when all dietary nitrogen was given in the form of amino acids [27].

Inhibition of enzymes involved in the synthesis of arginine would reduce arginine levels. However, it is believed that lysine does not have an effect on de novo synthesis of arginine, since a decrease in plasma arginine was also observed in animals that are unable to synthesize arginine and need to obtain all required arginine from their diet [25, 27].

Levels of arginine may be reduced when the amino acid is used for the synthesis of proteins. The most striking sign of arginine deficiency, however, is reduced growth (i.e. reduced synthesis of macromolecules) that is not caused by decreased food intake. Therefore it seems implausible that arginine deficiency is the result of an enhancing effect of lysine on protein synthesis.

In addition to arginine utilization for protein synthesis, the amino acid can also be used in the urea cycle for (1) the synthesis of urea, using the enzyme arginase and (2) the production of creatine in a reaction catalyzed by the enzyme arginine:glycine amidinotransferase (AGAT) (see Fig. 2). In multiple experiments, Jones et al. demonstrated that in chicks, lysine did not increase AGAT activity, nor that it increased creatine levels [27], thereby ruling out that lysine lowers plasma arginine levels by enhancing AGAT activity and creatine synthesis in chicks. On the other hand, the authors found that excess lysine was associated with increased arginase activity in chicks. This finding is in agreement with the results of Austic and Nesheim, who showed a dose dependent increase of arginase activity and urea production in chicks when fed excess dietary lysine [38], and higher arginase activity in chicks with a higher arginine requirement for growth [39]. However, the authors concluded that increased arginase activity is unlikely to be the only mechanism behind the arginine antagonism of lysine in chicks, since plasma arginine levels decreased before arginase levels increased [27]. Contrasting results were obtained in puppies, which showed, among others, decreased urea production and accumulation of ammonia upon excess dietary lysine [28]. If lysine would enhance arginase activity, catalyzing the reaction arginine to ornithine, higher urea levels may be expected. Arginase levels were indeed unaffected by the high concentration of lysine. Furthermore, no accumulation of ornithine in plasma or tissues of chicks or puppies was found, which would be expected if the activity of either arginase or AGAT was increased [27, 28]. To the best of our knowledge, a possible role in the lysine-arginine antagonism of nitric oxide synthase, catalyzing the production of nitric oxide from arginine, and arginine decarboxylase, catalyzing the synthesis of agmatine from arginine, has not been investigated.

Results from studies in rats and dogs do not support the hypothesis that high plasma lysine concentrations result in competition with arginine for reabsorption in the proximal tubules of the kidneys [28, 29]. Rats and dogs fed diets high in lysine excreted more lysine in their urine than arginine, both in absolute numbers and proportionally, when compared to urine samples from animals fed a basal diet. Furthermore, the absolute amount of arginine excreted was an insignificant portion of the amount of arginine that the animals consumed.

In conclusion, despite all efforts and after more than half a century of research, the precise mechanism of lysine-arginine antagonism remains poorly understood and may be different between species. Furthermore, there is no evidence for lysine-arginine antagonism in the cat.

#### Herpesvirus replication and arginine

The mechanism proposed by others by which lower arginine concentrations would reduce herpesvirus replication is rather simple: the virus needs a lot of arginine for protein synthesis and by restricting the supply of arginine, the virus cannot make sufficient viral proteins for the production of new virions. However, this proposed mechanism is based on two assumptions. It is assumed that (1) the virus needs more arginine than the cell can supply, and that (2) there is a means to limit intracellular arginine levels.

FHV-1 and HHV-1 need cellular arginine, as well as all other amino acids, for the synthesis of new virus particles. In tumor-derived cell lines cultured in arginine-free medium, HHV-1 was unable to replicate [14, 16], because its late stage proteins were not synthesized [40, 41]. In none of these studies, however, detailed information was provided on how the absence of arginine in the culture medium affected normal, non-viral, protein synthesis and cell growth, making it difficult to conclude that arginine supply was limiting for the virus. The effect of lysine supplementation in vivo is typically determined by measuring arginine levels in plasma, not intracellular. However, lowering the availability of intracellular arginine levels, and not plasma levels, is what, in theory, could prevent viral protein synthesis. Intracellular arginine levels are an order of magnitude higher than extracellular arginine concentrations [42–44], and even after culturing primary bovine aortic cells in arginine-free medium for 24 h, intracellular arginine concentrations were higher than normal plasma arginine concentrations [42]. These findings do not support the hypothesis that depletion of intracellular arginine can contribute to halting synthesis of viral proteins.

The block of HHV-1 replication at the level of late stage protein synthesis was observed in a cell line, whereas in primary endothelial cells intracellular arginine levels were higher than normal plasma concentrations after culturing in arginine-free medium. Jeney et al. investigated the replication kinetics of HHV-1 in arginine-free medium using three different tumor-derived cell lines and three different primary cells types [45]. While the virus was unable to replicate normally in the cell lines, replication of HHV-1 in all primary cell types was similar to the controls cultured in medium with a normal arginine concentration. It was proposed by the authors that intracellular arginine stores in cell lines are lower because of increased metabolic demands due to continuous growth of the cells [45, 46]. Indeed, tumor cells have an increased demand for arginine (reviewed in [47, 48]). In vitro studies performed by Scott et al. showed that arginine deprivation resulted in death of nearly all the different malignant cell cultures tested after several days. In contrast, primary cells survived for weeks in arginine deficient medium [49].

In conclusion, HHV-1 replication in primary cells was not affected by a lack of extracellular arginine, while results obtained from herpesvirus replication experiments in cell lines are likely to be less relevant because of the aberrant arginine requirements of these cells. Furthermore, the in vivo situation will not be as extreme as some of the conditions tested in the in vitro experiments, such as complete lack of extracellular arginine. Even when arginine levels in the blood can be lowered by lysine, blood circulation will still continuously supply arginine to the infected cells. Hence, it seems unlikely that it will be possible to reach a situation in vivo where arginine becomes limiting for HHV-1 or FHV-1 replication.

In the very first paper published on the topic of lysine supplementation and HHV-1 infection by Dr. Kagan, it was mentioned that viral proteins contain more arginine than the host proteins in the infected cell [50], referring to the work of Olshevsky et al., Spring et al. and Kaplan et al. [46, 51, 52] in the late 60’s and early 70’s. The availability of the genome sequences of human, cat and herpesviruses enables us to perform a much more detailed comparative analysis of arginine content in these organisms. Our analysis shows that the most arginine-rich HHV-1 protein is tegument protein US11, consisting of 19 % arginine. Interestingly, when we compared the human exome (protein coding part of the genome) with the HHV-1 exome, we found that human cells code for proteins with even higher arginine content than the virus (see Additional file 2). About 100 of the approximately 80,000 human proteins (length ≥ 100 amino acids) consist of about 20 % or more arginine (range of 19–36 %). However, on average, the HHV-1 exome contains more arginine (8.5 %) than the human exome (5.4 %). We are aware that this exome analysis does not necessarily reflect the proportion of arginine among the total protein pool in the cell, since the abundance of individual proteins varies. However, comparing the absolute fraction of arginine in the HHV-1 proteome with that of the human proteome is difficult since the exome provides qualitative data, not quantitative, and, to the best of our knowledge, quantitative proteomics data sets of uninfected and HHV-1 infected human cells are not available for analysis. It seems unlikely, however, that the fraction of arginine in the HHV-1 proteome is much different from that of its exome since the average number of arginine residues for capsid, tegument, envelope and the remaining proteins is 8.6, 9.0, 7.1 and 8.6 %, respectively (see Additional file 2).

**Fig. 3:**
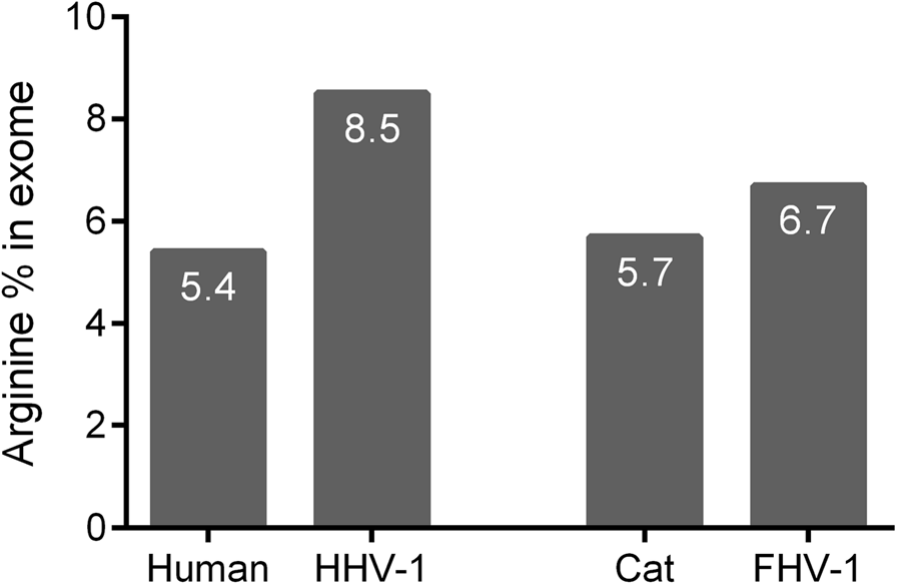
Arginine percentage of the exome in humans, human herpesvirus 1 (HHV-1), cats and feline herpesvirus 1 (FHV-1). Unlike HHV-1, FHV-1 does not require a lot more arginine for protein synthesis than its host

While the fraction of arginine in the cat exome is similar to that in the human exome (5.7 % in cats and 5.4 % in humans), the FHV-1 exome contains less arginine than the HHV-1 exome (6.7 % for FHV-1 and 8.5 % for HHV-1). The difference in arginine proportion between host and pathogen is thus smaller for cats and FHV-1 (5.7 and 6.7 %, respectively) than for humans and HHV-1 (5.4 and 8.5 %, respectively) (see Fig. 3), suggesting it is highly unlikely that replication of FHV-1 inside a cat’s cell will lead to a sudden increase in arginine demand.

#### Essentiality of arginine in cats

It has been demonstrated that excess dietary lysine does not affect plasma arginine levels in adult cats [32]. Furthermore, it seems that FHV-1 is less dependent on arginine than human herpesvirus 1 is (our data). Irrespectively, trying to lower arginine levels in the cat is undesirable.

**Fig. 4:**
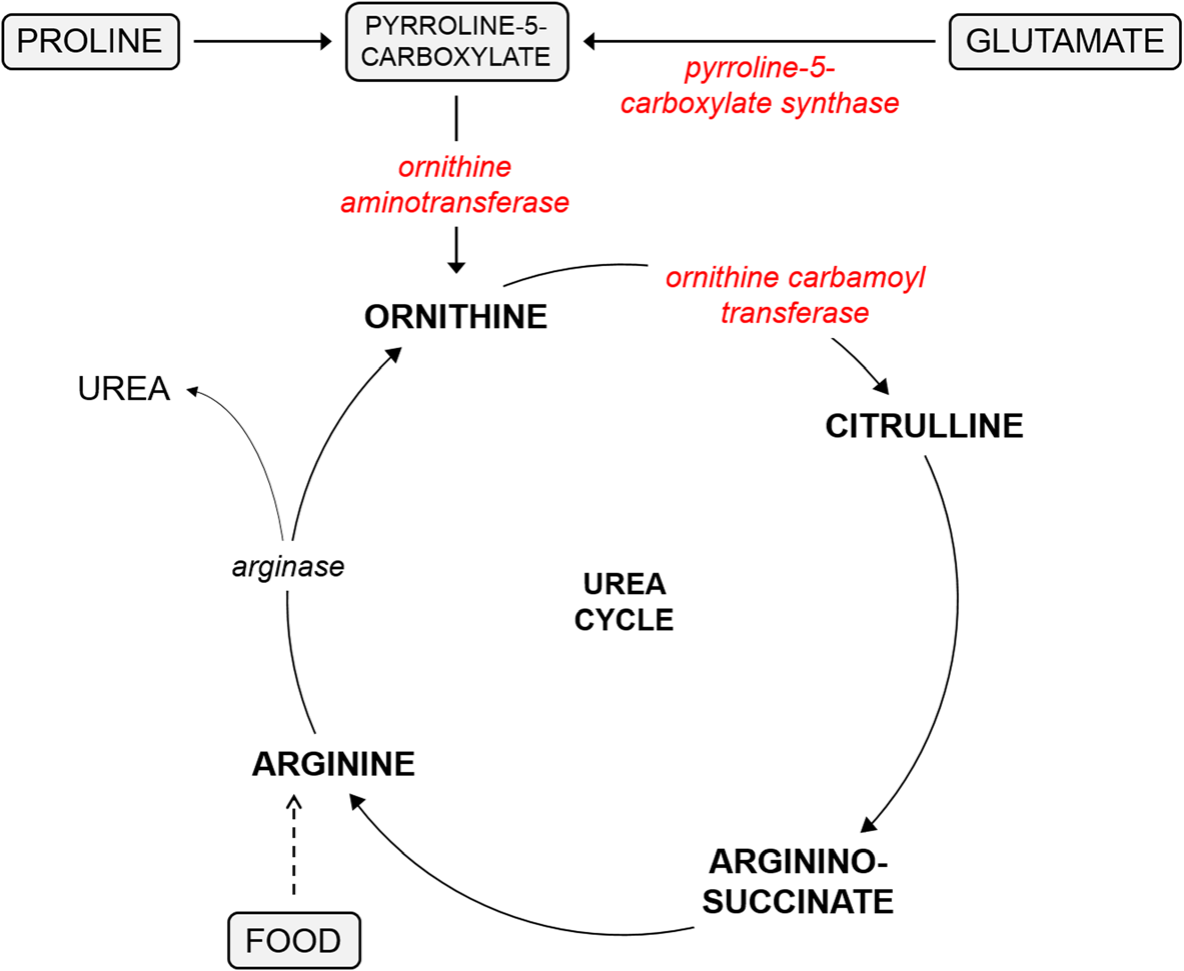
Simplified version of the urea cycle in the cat. The names of enzymes are italicized. Enzymes shown in red are present at low levels in cats, making cats dependent of arginine in their food

Arginine is an essential amino acid in cats. In fact, arginine is so critical for this species, no attempts should be made to restrict this amino acid in their diets. Humans fed a diet completely lacking arginine for 5 days showed no clinical signs and no toxic ammonium levels, demonstrating that we can synthesize arginine when needed [53]. Feeding cats a diet free of arginine or with low amounts of arginine, however, has severe consequences, among others rapid weight loss (100 g/day), complete refusal to eat and death as the result of ammonia intoxication [35, 54]. Because of the protein-rich diet of cats, large amounts of ammonium, a by-product of protein and amino acid metabolism, need to be excreted from the body, which in turn requires a lot of arginine [33]. Arginine is an essential component of the urea cycle, the pathway by which mammals get rid of ammonia (see Fig. 4). Urea is produced in the conversion of arginine to ornithine, by the enzyme arginase. Cats lack the ability to synthesize ornithine and hence also citrulline, because of relatively low activity of the enzymes pyrroline-5-carboxylate synthase and ornithine aminotransferase, that can generate ornithine from glutamate and proline [55, 56]. However, also when ornithine was added to an arginine-free diet, cats still lost weight [57], indicating that not enough citrulline can be made from ornithine, because of low levels of ornithine carbamoyl transferase [58]. Arginine can be synthesized from citrulline, and cats fed an arginine-free diet supplemented with citrulline did not show any clinical signs [57]. The synthesis of arginine from citrulline is limited in cats because of low plasma citrulline levels. The inability to synthesize any of these other urea cycle intermediates make cats completely dependent of arginine in their food. Therefore, lowering plasma arginine, either by restricting arginine in the diet or via other indirect ways, is undesirable, especially when lysine (amino acid) is added to their diet.

### In vitro studies

Cell cultures are an excellent tool to test for antiviral activity of molecules. Although the results do not always translate well into in vivo systems, interesting data can be obtained in a relatively timely manner, experiments are relatively inexpensive and ethical issues are avoided in most cases. Indeed, the outcome of several in vitro experiments with human herpesvirus stimulated clinical research with patients suffering from herpes labialis or genitalis, but also led to similar experiments with feline herpesvirus 1 [7] and experiments with cats [10, 11, 13]. In this section, we describe the experiments that were performed to evaluate the effect of lysine on the in vitro replication of HHV-1 and FHV-1, and their results.

#### Human herpesvirus 1

It was Tankersley, in 1964, who for the first time described that human herpesvirus 1 requires the amino acid arginine for its replication in vitro [16]. His experiments were performed using an esophageal epithelial cell line of human origin. Tankersley also found that lysine was not required for HHV-1 replication, and that lysine (70–420 μg/ml) had an inhibitory effect when the cells were cultured in Eagle’s medium (EMEM; 105 μg arginine/ml [59]). The inhibition, however, although significant, was not dose dependent and not complete.

Cell lines are homogenous and easy to maintain, but are often derived from tumors and have adapted to growth in culture. Primary cells, obtained directly from tissue, are more difficult to work with, but do better resemble in vivo conditions. A few years after Tankersley published his results, Jeney et al. was able to replicate Tankersley’s findings, studying HHV-1 replication in arginine-free medium using 3 different cell lines (as also discussed in the Mechanism section) [45]. However, when primary cells were used (primary monkey kidney cells, primary chick embryo fibroblasts and human embryonic fibroblasts) the authors discovered there was no difference in HHV-1 replication between cells cultured in arginine-free or control medium.

Many years later, in 1981, Griffith et al. again showed that arginine is required for HHV-1 replication in kidney epithelial cells of monkey origin (BSC1 cell line), and that lysine can inhibit HHV-1 replication [14]. However, again, inhibition was not dose dependent and not complete. Experiments were done using 7 different, all subnormal, concentrations of arginine in the medium, ranging from 0 to 25 μg per ml. Lower arginine levels were associated with a stronger inhibitory effect of lysine. At the highest arginine concentration tested (25 μg per ml), no inhibition with any of the lysine concentrations (50–400 μg/ml) was observed anymore, whereas Tankersley reported inhibition with similar lysine concentrations in combination with higher arginine levels [16]. The ratio lysine to arginine did not seem to be of importance. While complete inhibition was observed by the authors at lysine to arginine ratios of 10, 13, 10 and 8 at arginine concentrations of 5, 7.5, 10 and 12.5 μg/ml, respectively, no inhibition was seen at a lysine to arginine ratio of 16 when the arginine concentration was 25 μg/ml. Infected cells were cultured until the control samples (105 μg arginine and 58 μg lysine per ml) died as the result of the viral replication. No information was provided about cell viability of the infected cells cultured with subnormal arginine concentrations.

Park et al. studied if, among others, lysine was able to block reactivation of latent HHV-1 in primary cells [15]. Trigeminal ganglia mouse cells were cultured in EMEM. These primary nerve cells may better resemble the in vivo conditions than the cell lines used by Tankersley and Griffith, most certainly when studying viral latency and reactivation. Although about 2 log inhibition of HHV-1 replication was seen directly after the cells were cultured for 1–3 days in medium containing 200 μM (30 μg/ml) lysine, titers returned to similar levels compared to the control samples within 1–2 days after medium change to normal EMEM. The authors concluded that lysine supplementation was unable to prevent viral reactivation. It is difficult to interpret the results of this study since the lysine concentration in the control medium, EMEM, was 400 μM [59]. According to the authors, 200 μM (30 μg/ml) lysine was a high concentration of the amino acid [15, 60], but it is less than the higher concentrations used in previously published in vitro studies (70–420 μg/ml [16] and 50–400 μg/ml [14]). Unfortunately, Dr. Park was not able to clarify this issue (personal communication).

#### Feline herpesvirus 1

Two in vitro studies examined the effect of lysine on the replication of feline herpesvirus. In the study by Maggs et al. [7] the feline kidney cell line CRFK was used. The authors showed that FHV-1 was unable to replicate in arginine-free medium. Although the authors claimed that culturing the CRFK cells in 200–300 μM (35–55 μg/ml) lysine inhibited viral replication, none of these results were statistically significant. In fact, these lysine concentrations increased viral replication when the medium contained 5 μg arginine per ml instead of 2.5 μg/ml. Unfortunately, the authors did not test the effect of lysine in culture medium resembling the in vivo physiology of cats more closely, since only subnormal arginine concentrations were used. The results from their own study indicate that they used subnormal conditions, since at 2.5 and 5 μg arginine/ml, in the absence of lysine in the medium, the viral titer was less than 50 % of the controls (DMEM medium, containing 146 μg lysine and 84 μg arginine per ml). Indeed, normal plasma lysine and arginine levels in healthy cats are 12–15 μg/ml and 17–23 μg/ml, respectively [11, 13], and with lysine supplementation, plasma lysine levels reached as high as 44–70 μg/ml [10, 11, 32]. Other in vitro studies with herpesvirus used arginine concentrations > 100 μg/ml [15, 16] and up to 25 μg/ml [14]. In the latter no inhibition of lysine was observed anymore when the arginine concentration was 25 μg/ml.

Cave et al. acknowledged the concerns addressed above and the goal of their study was to determine the effect of lysine on FHV-1 replication in vitro under conditions that better mimic the in vivo situation and that support normal cell growth [8]. The authors used arginine- and lysine-free medium and supplemented the medium with different amounts of the two amino acids: 6, 12, 18 and 126 μg arginine per ml and 10, 20, 40, 80, 160 and 320 μg lysine per ml. The same cell line was used as in the study of Maggs et al. and conditions were tested using two different virus to cell ratios (multiplicity of infection of 0.1 and 1). The authors found that viral replication did not differ between cells cultured in media with different concentrations of lysine and concluded that lysine does not inhibit replication of FHV-1 in vitro.

In summary, the results from the in vitro studies with HHV-1 are inconsistent [14, 16] and sometimes unclear [15]. In two studies, one with HHV-1 [14] and one with FHV-1 [7], the effect of lysine was only studied with arginine concentrations that did not support normal cell growth or maintenance. The observed inhibition of viral replication may have been the result of increased cell death, rather than an inhibitory effect of lysine. Dr. Cave mentioned that at the arginine concentrations used by Maggs et al., cell viability was only 10 % as compared to the controls [8]. Lysine did not inhibit FHV-1 replication at any of the tested lysine concentrations when arginine levels allowed normal cell growth. It should be noted that cell lines, and not primary cells, were used in all but one studies. One study that was not cited by any of the authors was the work by Jeney et al., who showed that, in contrast to in cell lines, HHV-1 replicated in primary cells cultured in arginine-free medium, with kinetics similar to cells grown in control medium [45].

Based on the results described above, we conclude that lysine does not inhibit replication of FHV-1 in vitro. The results for FHV-1 are summarized in Table 1.

**Table 1:** Summary of studies that investigated the effect of lysine on FHV-1 replication in vitro, or on the prevention or treatment of FHV-1 infection and its disease manifestations in cats

Study	N	Outcome	
Maggs et al. (2000) [7]	N/A	Lysine did not reduce the replication of FHV-1 in vitro.	−
Stiles et al. (2002) [13]	8	Lower mean clinical score for experimentally induced conjunctivitis during days 5–15 in a group of 4 cats receiving supplemental lysine when compared to 4 controls.	+
Maggs et al. (2003) [10]	14	Supplementing lysine did not reduce the chance of developing conjunctivitis or reduce its severity.	−
Maggs et al. (2007) [11]	50	A lysine-rich diet did not decrease the frequency or severity of ocular or upper respiratory disease in cats housed under shelter-like conditions.	−
Rees et al. (2008) [12]	291	Supplementation of lysine did not reduce the number of shelter cats developing upper respiratory infections or conjunctivitis.	−
Drazenovich et al. (2009) [9]	123	Lysine supplementation did not lower the incidence of ocular or upper respiratory disease in shelter cats.	−
Cave et al. (2014) [8]	N/A	Lysine did not inhibit replication of feline herpesvirus 1 in vitro.	−

N, sample size; N/A, not applicable

### Clinical studies

#### Clinical trials on humans infected with human herpesvirus 1

Human herpesvirus 1 and feline herpesvirus 1 are members of different genera (Simplexvirus and Varicellovirus, respectively), but both belong to the alphaherpesvirinae, a subfamily of the herpesviruses. Publications claiming a positive effect of lysine supplementation on the treatment or prevention of herpes labialis or genitalis outbreaks in humans were at the basis of research on the efficacy of lysine supplementation in cats infected with FHV-1, both in vitro [7] and in vivo [10, 13]. In this section we will therefore critically evaluate all clinical studies investigating the efficacy of lysine supplementation in humans infected with HHV-1, in a chronological order.

The first publication suggesting a possible role for lysine in the treatment of herpetic lesions was a letter written by Dr. Kagan [50] in 1974. Four years later a first study appeared, under the leadership of Dr. Kagan, in which the authors claimed a beneficial effect of lysine supplementation on the treatment and prevention of herpes simplex outbreaks [61]. However, this study was not blind (patients knew which medication they received and what the goal of the study was) and there was no control group receiving a placebo. These types of studies are of no scientific value. Indeed, shortly after this study by Griffith et al. was published, a group in Denmark reported that they were unable to replicate these findings. This group (Milman et al.) investigated if lysine supplementation was effective to treat herpetic lesions. The results of this randomized, double-blind, placebo-controlled case–control study in which 119 patients were followed for almost a full year showed there was no difference in the recurrence of herpes outbreaks between the group receiving 1000 mg lysine per day (500 mg twice a day) and the group receiving a placebo [20]. Patients were instructed to start taking their pills (for the duration of about a week) when they first started noticing symptoms. The authors argued that they may have missed an effect of lysine in their study because virus replication may have started before the first symptoms were noticed. But also after 65 patients were given 1000 mg lysine (500 mg twice daily) or a placebo every day for a period of almost half a year in a randomized, double-blind, cross-over study (2 × 12 weeks), no prophylactic effect of lysine on the recurrence of herpes simplex labialis was observed [21].

Walsh et al. claimed that lysine reduced the number of attacks and shortened healing time [62]. This article, however, describes the results of a questionnaire given to people who bought lysine in a nutrition store. It was uncontrolled and therefore, like other, similar reports [63, 64], results are not reliable. These papers cannot be used to help determine the efficacy of lysine on the prevention or treatment of herpetic lesions. Despite the poor quality of these articles, some authors, unfortunately, still cite these publications, sometimes selectively, using their untrustworthy results to support their claims [7, 10, 13, 14, 18, 19, 23, 61–63, 65].

A small (*n* = 20), but well controlled (randomized, double-blind, placebo-controlled case–control) study [17] in which the effect of 1200 mg lysine per day on recurrence, duration and severity of herpetic lesions was investigated for 4–5 months, confirmed the negative findings of Milman et al. [20, 21]. A study performed by McCune et al. [19] had a size (*n* = 20) and design somewhat similar (double-blind, placebo-controlled cross-over) to the study performed by DiGiovanna et al. [17]. The authors did not find a positive effect of lysine on healing rate, but did describe that oral ingestion of 1250 mg lysine daily for a period of 24 weeks lowered the number of recurrences when lysine was taken as compared to when the patients took a placebo.

Thein and colleagues [23] were the first to study if there was a correlation between both plasma lysine and arginine levels, and the number of herpetic lesions. In this double-blind, placebo-controlled cross-over study (*n* = 26) that lasted for 1 year, it was attempted to control for dietary factors to determine efficacy of lysine supplementation, as was also suggested by Algert et al. [66]. In all studies, dietary changes were suggested or recommended, but diet has never been well controlled for. Thein et al. found a reduction in the number of lesions, but it was independent of the treatment type (1 g lysine/day or placebo) [23]. It was hypothesized that lysine may be effective only when a certain minimum level of lysine in the plasma is reached. Algert’s paper described that there is no difference in dietary intake for lysine and arginine between a group of patients with initial or recurrent herpes genitalis infections and a control group, although it is not clear how many persons in the control group were latently infected [66]. In addition, the authors mention that the American diet is rich in lysine (6–10 g per day), which makes deficiencies rare since the recommended intake is about 2 g per day [67].

In two randomized, double-blind, placebo-controlled case–control studies, Simon et al. [22] (1 g/day for 3 months, *n* = 31) and Griffith et al. [18] (3 g/day for 6 months, *n* = 52) claimed positive results of lysine supplementation. However, both studies compared the number of herpetic outbreaks with self-reported predicted recurrence rates based on the patient’s history, thereby introducing unnecessary bias (response from DiGiovanna et al. in [22]). When the number of outbreaks was compared between the group that received 3000 mg lysine daily and the group receiving a placebo, no difference was seen [18]. No new study results on this subject were published after this publication in 1987.

In addition to the small sample sizes, poor study designs and improper analyses, we have other concerns that need to be addressed. Several of the researchers who published about lysine and HHV-1 held a position at a pharmaceutical company manufacturing lysine supplements (Lilly and GNC), and we therefore feel there was a conflict of interest. All their publications described a positive effect of lysine supplementation [14, 18, 61, 62]. Furthermore, there may be a publication bias, since journals are less willing to reports negative findings (e.g. a study describing the absence of an effect) than studies with statistically significant results [68]. There may have been other research groups that were unable to demonstrate the efficacy of lysine, whose results were not published.

Based on the results of the 7 clinical trials that were included for qualitative analysis, we conclude that the claim that lysine supplementation is effective for the prevention or treatment of herpetic lesions in humans cannot be supported by scientific evidence. This view is shared by many scientists and medical doctors since lysine supplementation is not part of the guidelines for the treatment or prevention of herpes labialis or genitalis [69–71]. Very recently, after conducting a systematic review evaluating different interventions for the prevention of herpes labialis, Chi et al. also concluded there is no evidence for the efficacy of lysine [72]. In addition, as discussed earlier, there is no evidence that lysine-arginine antagonism occurs in humans [31]. Therefore, studies into the effect of lysine supplementation in cats have been based on the incorrect premise of its success in HHV-1 studies and clinical trials.

#### Clinical trials on cats infected with feline herpesvirus 1

The first study investigating the effect of lysine supplementation on the clinical course of cats suffering from FHV-1 infection was published in 2002 by Stiles et al. [13]. This blinded, randomized and placebo-controlled study with a total study population of only 8 cats, specifically studied the effect of lysine supplementation on experimentally induced conjunctivitis. For a period of 3 weeks, the cats received 1000 mg lysine daily or a placebo (lactose), beginning 6 h prior to inoculation with the virus. Although higher in the lysine group, plasma lysine increased significantly during the study period, in both the lysine and the placebo group. Contrary to the authors’ hypothesis, compared to baseline values, plasma arginine levels were significantly increased as well, both in cats receiving supplemental lysine and in cats receiving the placebo. Arginine levels were never significantly different between the two groups. Irrespective of the similar and increased plasma arginine levels, the authors reported a statistically significant lower mean clinical score for conjunctivitis for the lysine group when compared to the placebo group. However, this was only observed for the period of day 5–15, not for the entire study period of 21 days. In the third week of the study, there was no difference between the two groups of cats, and time to resolution also was the same. Finally, viral shedding was not different in the lysine group when compared to the controls.

In a second study, with a comparable small study population, Maggs et al. fed 7 randomly assigned cats 400 mg supplemented lysine per day and 7 other cats were used as controls [10]. All cats were inoculated with FHV-1 about 5 months prior to the study period of 30 days, and at day 15 of the study corticosteroids were administered to the cats to reactivate the virus. Plasma lysine levels doubled in the treatment group, but the increase lasted for only 2–3 h before levels were back to normal again. Lysine supplementation did not affect plasma arginine levels. During the first 15 days of the study, none of the cats showed any clinical signs associated with active FHV-1 replication. Nevertheless, the authors observed a higher viral shedding rate (defined as the number of days where FHV-1 DNA was detected in the fluid between the eyeball and eyelid, known as the conjunctival fornix) in the control group as compared to the cats receiving lysine supplementation. The number of cats or eyes shedding virus did not differ between the groups. The authors mentioned that stress may have induced reactivation of the virus. After corticosteroid administration, cats in both groups developed conjunctivitis. There was no difference between the 2 groups in the number of cats affected or in the course of the inflammation. All cats recovered without treatment and time to recovery was not different between the two groups. The difference in viral shedding rate observed during the first 15 days of the study was no longer seen in the second part of the study.

In 2007 Maggs et al. studied the effect of lysine-enriched cat food on the clinical course of ocular disease and upper respiratory disease caused by FHV-1 in a group of 50 cats housed under conditions comparable to feline shelters [11]. Two groups were made, stratified for gender and FHV-1 serological status (12 were seropositive in each group of 25). For a period of 52 days, one group was fed food containing 51 g lysine per kg diet, while the other group received a basal diet containing 11 g lysine per kg diet (control group). The researchers were blinded to which group of cats received the lysine-enriched food. Arginine content of the food did not differ between the two groups (12–13 g per kg food). Male and female cats in each group were caged separately.

Plasma lysine and arginine levels were determined at baseline, during and at the end of the study. However, only 3 measurements were taken, which does not allow extrapolation of the data to the complete study period (52 days). A previous study by Maggs et al. [10] showed that plasma lysine was elevated only 2–3 h after ingestion. Unfortunately, no information was given about the moment of blood collection of the 3 samples in this study. At day 17 and at the end of the study at day 52, plasma lysine levels in the lysine group were elevated compared to baseline values, and 1.5–3 times higher than in the control group. Contrary to previous studies, the authors reported a decline of plasma arginine levels. However, this was observed in both groups, albeit a significantly stronger decrease in the lysine group (60 nmol/ml at the end of the study; a 40 % reduction compared to baseline values) than in the controls (80 nmol/ml; a 25 % reduction). Since plasma lysine did not increase in the control group, the decline in plasma arginine levels cannot be attributed to lysine supplementation. Contradictory to the previous, smaller studies, the authors found that ocular and upper respiratory disease were more frequent among cats receiving the lysine-rich food (88 %) as compared to the control group (60 %), and clinical signs were also more severe. The authors speculated that aggression among the male cats in the lysine group may have induced additional stress, possibly reactivating the virus. However, the clinical severity score for female cats in the lysine group was higher at all 15 measuring points throughout the study when compared to females fed the basal diet, and this difference approached statistical significance (*P* = 0.08). The authors did not compare the complete (male and female) control group with the females only in the lysine group. Seven cats seroconverted for FHV-1 during the study, 6 in the lysine group and 1 in the control group (which had a lower titer than all 6 cats in the lysine group, and undetectable viral DNA). FHV-1 was detected in conjunctival fluid in 7 cats on 13 occasions: 1 cat in the control group on 1 occasion and 6 cats in the lysine group on 12 occasions.

In short, we can conclude that feeding lysine-enriched food to cats was unable to prevent or reduce viral shedding, seroconversion or decrease the frequency or severity of clinical signs caused by FHV-1.

Rees et al. also found no beneficial effect of oral supplementation of 500 mg lysine per day on the prevention of upper respiratory infections and conjunctivitis in cats in a shelter [12]. In this blinded study, cats were randomly assigned to either a lysine group (250 mg daily for kittens and 500 mg for older cats) or a control group. The lysine powder was mixed with a small amount of canned food, to make administration not stressful for the cats. Food intake between the groups was the same. A total of 291 cats participated in the study, which lasted from the day they were brought into the shelter till the day they were adopted. This large study population would allow the investigators to even find a small effect of lysine. Lysine supplementation in cats showing clinical signs of upper respiratory disease or conjunctivitis was discontinued. These cats were isolated from the other cats and treated with antibiotics. In the lysine group, 37 % (53 out of 144) of the cats developed clinical signs, compared to 34 % (50 out of 147) in the control group. There was also no difference between the groups in the number of days the cats stayed free of clinical signs (13 days in both groups) or the time needed to treat till declared healthy.

The most recent clinical trial studying the efficacy of lysine supplementation on ocular or upper respiratory disease in cats was published in 2009 by Drazenovich et al. [9]. This work confirmed earlier negative findings by Maggs et al. [10, 11] and Rees et al. [12]. Shelter cats were fed either a basal diet (17 g lysine/kg food) or a lysine-enriched diet (57 g lysine/kg food), with equal arginine content (19 g/kg food). Although 261 cats were enrolled in the study, many were lost to follow-up. One hundred and twenty-three cats were followed for 1 week (62 in the lysine and 61 in the control group), and at the end of the 4 week study period the total study population size was 47. The study was randomized and blinded. Plasma lysine and arginine levels were measured at the start and at the end of the study. The authors did not mention how many hours after a meal the blood samples were taken. Plasma lysine levels were significantly higher at the end of the study in the lysine group, but, as expected, not in the control group. Arginine levels were not affected by the diet. The authors concluded that cats in the lysine group were more likely to develop moderate to severe signs of disease than cats fed the basal diet, and found that more cats in the lysine group tested positive for FHV-1 than in the control group.

Based on the results described above (summarized in Table 1), we conclude that lysine supplementation in cats is not effective for the prevention or treatment of ocular or upper respiratory disease caused by FHV-1, and may even have an enhancing effect on viral replication.

## Conclusion

**Fig. 5:**
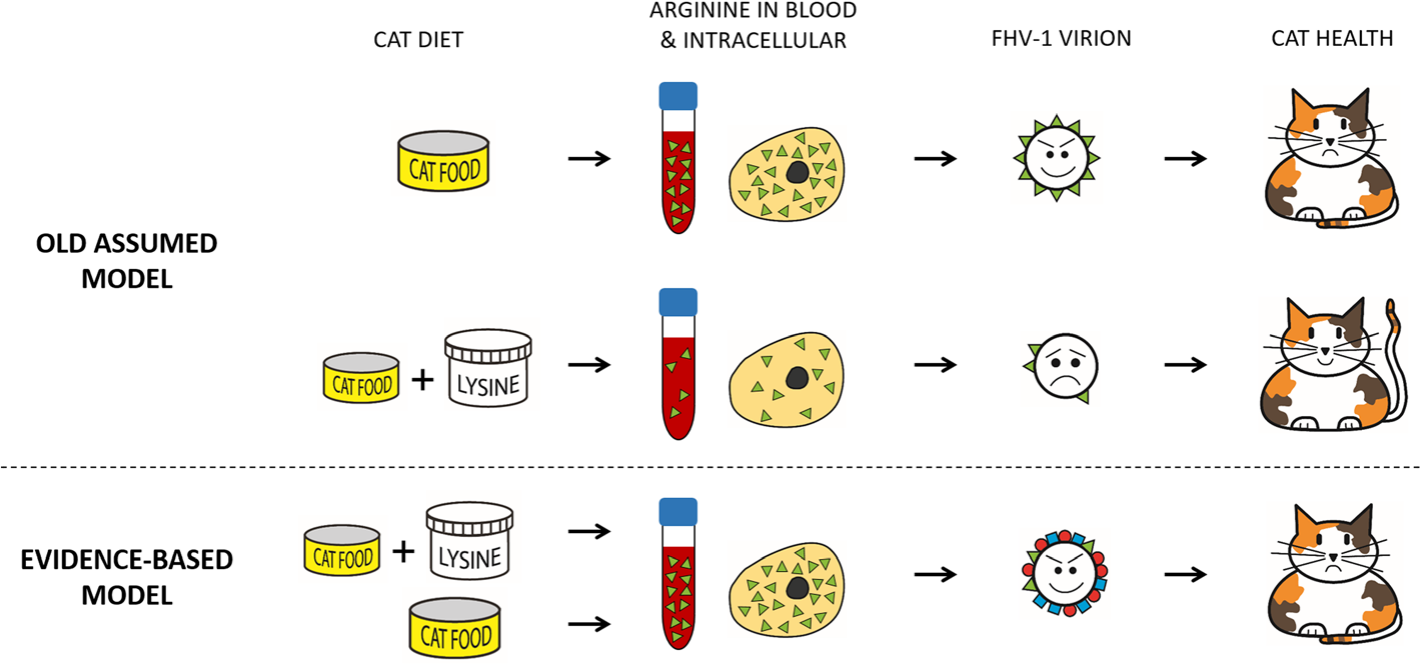
Schematic summary of our conclusion. *Top*. In the old model [10, 11, 13], it was assumed that supplementing cat food with lysine would lower plasma and tissue arginine (green triangles) to such low concentrations that FHV-1 would be unable to synthesize enough of its arginine-rich proteins. *Bottom*. In our evidence-based model, lysine supplementation does not lower arginine levels, and the synthesis of feline herpesvirus 1 proteins, which are not excessively rich in arginine (green triangles on virion), is not inhibited

Taking all results discussed in this systematic review together, we conclude that lysine supplementation does not have an inhibitory effect on FHV-1 replication in the cat. The scientific data do not support lysine supplementation or additional research with cats, as has been advocated by some (Maggs [4] and Dr. Stiles, personal communication). In contrast to the old, assumed model [10, 11, 13], we propose a new, evidence-based model in which arginine levels and viral replication are not influenced by lysine supplementation (see Fig. 5). Based on the complete lack of scientific evidence for the efficacy of lysine supplementation, we recommend an immediate stop of lysine supplementation for cats. Lysine supplementation is not effective to prevent cats from becoming infected with FHV-1, it does not decrease the chance of developing clinical signs related to active FHV-1 infection, and it does not have a positive effect on the clinical course of its disease manifestations. In fact, results from two clinical trials with cats even suggest that excess dietary lysine may have an enhancing effect on FHV-1 replication. Positive findings, either for HHV-1 or FHV-1, were the result of poor study design and could not be replicated in well-controlled, larger studies. Furthermore, the proposed mechanism of action of lysine-arginine antagonism does not work in cats and its result, lowering arginine levels, would be highly undesirable. The dominant arguments that form the basis of our conclusion on are listed in Table 2.

**Table 2:** Reasons why cats should not receive lysine supplementation for the treatment or prevention of FHV-1 infection

• Clinical trials with cats failed to show efficacy of lysine supplementation.
• In vitro studies with FHV-1 did not show an inhibitory effect of excess lysine on viral replication.
• Lysine itself does not have antiviral properties, but was believed to act by lowering arginine levels (lysine-arginine antagonism). In cats, excess lysine is unable to lower plasma arginine levels.
• There are no studies showing that intracellular arginine can be lowered to levels that will specifically prevent the synthesis of FHV-1 proteins. Furthermore, it is unlikely that FHV-1 replication in the cat’s cell specifically increases the cellular demand for arginine.
• Arginine is an essential amino acid for cats. Cats deficient in arginine will die as the result of hyperammonemia. No attempts should be made to lower arginine levels in cats, especially not when adding lysine (amino acid) to their diet.
• Lysine supplementation is not effective for the treatment or prevention of herpetic lesions in humans infected with human herpesvirus 1.

The best way to prevent reactivation of the virus is to minimalize physical and mental stress for the cat. Cats suffering from an active FHV-1 infection may be given extra palatable food and some extra love and attention. When signs of active infection present, it is recommended to have the cat seen by a veterinarian.

## Additional files

Additional file 1:
**PRISMA checklist.** (DOCX 25 kb) Additional file 2:
**Comparative analysis of arginine content of human, cat, HHV-1 and FHV-1 exome.** Arginine percentage of human, cat, HHV-1 and FHV-1 proteins. Protein name, protein length in amino acids, the number of arginine residues in the protein and the percentage of arginine in the protein are shown in columns A, B, C and D, respectively. Each data set is sorted on arginine percentage. For human and feline herpesvirus 1, structural proteins are classified based on Genbank annotations (column E). (XLSX 5460 kb)

## Abbreviations

AGAT: arginine:glycine amidinotransferase
EMEM: Eagle’s minimum essential medium
FHV-1: feline herpesvirus 1
HHV-1: human herpesvirus 1
